# The Pleiotropic Immunomodulatory Functions of IL-33 and Its Implications in Tumor Immunity

**DOI:** 10.3389/fimmu.2018.02601

**Published:** 2018-11-13

**Authors:** Claudia Afferni, Carla Buccione, Sara Andreone, Maria Rosaria Galdiero, Gilda Varricchi, Gianni Marone, Fabrizio Mattei, Giovanna Schiavoni

**Affiliations:** ^1^National Center for Drug Research and Evaluation, Istituto Superiore di Sanità, Rome, Italy; ^2^Department of Oncology and Molecular Medicine, Istituto Superiore di Sanità, Rome, Italy; ^3^Department of Translational Medical Sciences and Center for Basic and Clinical Immunology Research (CISI), University of Naples Federico II, Naples, Italy; ^4^WAO Center of Excellence, Naples, Italy; ^5^Institute of Experimental Endocrinology and Oncology “Gaetano Salvatore”, National Research Council, Naples, Italy

**Keywords:** IL-33, cancer, immune cell subsets, tumor immunology, IL-33 isoforms

## Abstract

Interleukin-33 (IL-33) is a IL-1 family member of cytokines exerting pleiotropic activities. In the steady-state, IL-33 is expressed in the nucleus of epithelial, endothelial, and fibroblast-like cells acting as a nuclear protein. In response to tissue damage, infections or necrosis IL-33 is released in the extracellular space, where it functions as an alarmin for the immune system. Its specific receptor ST2 is expressed by a variety of immune cell types, resulting in the stimulation of a wide range of immune reactions. Recent evidences suggest that different IL-33 isoforms exist, in virtue of proteolytic cleavage or alternative mRNA splicing, with potentially different biological activity and functions. Although initially studied in the context of allergy, infection, and inflammation, over the past decade IL-33 has gained much attention in cancer immunology. Increasing evidences indicate that IL-33 may have opposing functions, promoting, or dampening tumor immunity, depending on the tumor type, site of expression, and local concentration. In this review we will cover the biological functions of IL-33 on various immune cell subsets (e.g., T cells, NK, Treg cells, ILC2, eosinophils, neutrophils, basophils, mast cells, DCs, and macrophages) that affect anti-tumor immune responses in experimental and clinical cancers. We will also discuss the possible implications of diverse IL-33 mutations and isoforms in the anti-tumor activity of the cytokine and as possible clinical biomarkers.

## Introduction to IL-33 biology

Interleukin-33 (IL-33) is a cytokine member of IL-1 family, including IL-1α, IL-1β, IL-18, and IL-1Ra that are related to each other by receptor structure and signal transduction pathways. These cytokines share a conserved structure of β-trefoil fold comprised of 12 anti-parallel β-strands that are arranged in a three-fold symmetric pattern. The β-barrel core motif is packed by various amounts of helices in each cytokine structure (1). IL-33 was initially described in 2003 by Girard's group as a nuclear protein abundantly expressed in high endothelial venules (HEVs), specialized blood vessels that mediate the entry of lymphocytes into lymphoid organs and therefore named “nuclear factor from high endothelial venules” (NF-HEV) (2).

It is now known that IL-33 is a chromatin-associated nuclear cytokine *in vivo* through chromatin-binding motif within its N-terminal nuclear domain, suggesting that nuclear localization and binding to histones are important for IL-33 function and regulation (3). Nuclear IL-33 can function as a transcriptional repressor when overexpressed in transfected cells, although there is still no direct evidence that endogenous nuclear IL-33 regulates gene or protein expression (4). IL-33 is constitutively expressed in different human and mouse tissues in the steady-state, including epithelial, endothelial, fibroblast-like cells, and myofibroblasts and its expression can be increased during inflammation (2, 5). After cell stress or necrosis, IL-33 is released into the extracellular space and functions as an endogenous danger signal that alerts the immune system of tissue damage during trauma or infection. Indeed, IL-33 is considered an “alarmin” able to activate different actors of the innate immune system, mediating a variety of immune reactions including anti-cancer immune responses (6). Here, we will review the biological role of IL-33 affecting immune responses with particular emphasis on anti-tumor immunity.

### IL-33 isoforms

Similar to IL-1β and IL-18, IL-33 is synthesized in a full-length form (amino acids 1–270) that is found in the nucleus, in the cytosol and outside the cell. As IL-1β and IL-18, IL-33 is cleaved intracellularly by the enzyme caspase-1 before release outside the cell. This process requires the NLRP3 inflammasome, which can be activated in response to endogenous and exogenous danger signals. This NLRP3 inflammasome leads to Caspase-1 activation and, in turn, to IL-33 processing and release (7). When cells undergo necrosis or injury, full-length IL-33 is released in the extracellular space where it is cleaved by inflammatory proteases. During apoptosis, a process that does not trigger inflammation *in vivo*, IL-33 is cleaved and inactivated by endogenous caspases (8–10). Processing by apoptotic caspases is an important regulatory mechanism that limits or suppresses the pro-inflammatory properties of IL-33 during homeostatic cell turnover. Another regulatory mechanism limiting IL-33 activity is oxidation. Extracellular IL-33 is susceptible to cysteine oxidation that leads to the formation of disulphide bridges, resulting in conformational changes that inhibit the binding to ST2 receptor, thus rapidly inactivating IL-33 following allergen exposure (11).

**Figure 1:**
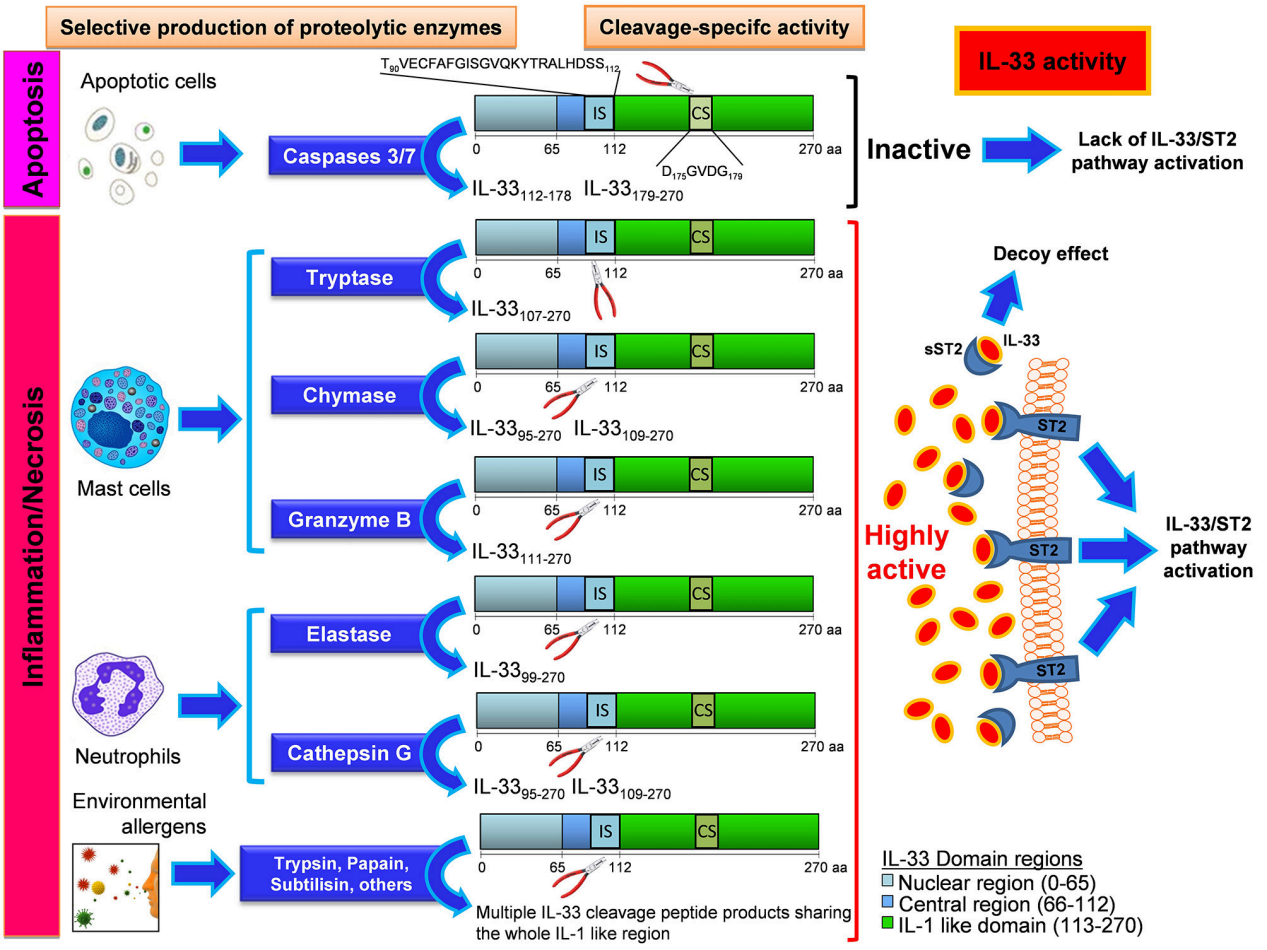
Mechanisms and effects of the enzyme-specific IL-33 cleavage. Biological events such as apoptotic stress, Inflammation, and necrosis can differentially generate various IL-33 protein variants with high biological activity or no activity depending on the enzyme produced by the cells. Apoptotic cells enable the production and release of caspases 3 and 7, that cleaves IL-33 in the caspase site (CS) generating inactive fragments of IL-33 by disruption of some IL-1 like functional domains close to CS. The insurgence of inflammation or necrosis process leads to the local recruitment of mast cells and neutrophils, the main effectors of inflammatory processes. When these cells reach the inflammed site, they produce, and release enzymes that cleave the IL-33 protein at the Inflammatory site (IS) inside the central region. These cleavage-specific enzymes allow the production and release of highly active forms of IL-33, capable to stably bind the ST2 receptor (ST2) on a plethora of ST2-expressing cells. In other cases this active IL-33 does bind to the soluble form of its receptor (sST2), which acts as a decoy receptor. In this latter event the effect of IL-33 will be suppressed by the formation of an sST2/IL-33 decoy complex. When no apoptotic nor inflammatory enzymes are produced, an uncleaved form of IL-33 is released, with a very low biological activity compared to that showed by IL-33 cleavage products originated by neutrophil and mast cell-derived enzymatic cutting. When the IL-33 is cleaved by certain environmental allergens, their enzymatic activity at the IS site gives rise to multiple peptide products sharing the whole IL-1 like region of IL-33, that does retain the ability to bind ST2. The IL-33 cleavage products herein shown are all equipped with the ST2 binding sequence (inside the IL-1 like region). The secondary fragments lacking the ST2 binding sequence and generated during the cleavage reaction are not depicted and have no effect on IL-33/ST2 binding.

Recent studies have demonstrated the existence of several human full-length active mRNA splice variants dependent on both the cell type expressing IL-33 and the pathological condition and triggered by diverse stimulations during immune responses (12–14). Of note, inflammatory proteases from neutrophils (proteinase 3, elastase, and cathepsin G) (15), mast cells (chymase, tryptase, and granzyme B) 16, and environmental allergens (17) can process full-length IL-33 into shorter mature forms (18–21 kDa) whose biological activity is 10- to 30-fold more potent than the full-length form (see Figure 1). The mature form does not translocate into the nucleus because it lacks the nuclear localization signal found in full-length IL-33 (18, 19). Proteolytic cleavage of IL-33 was shown to induce allergic inflammation *in vivo* (17) highlighting a novel mechanism by which inflammatory and environmental proteases can amplify allergic inflammation. Of interest, isoform variants as well as cleavage by endogenous and exogenous proteases has been described also for other epithelial-derived cytokines, such as thymic stromal lymphopoietin (TSLP), resulting in pleiotropic functions in health and disease (20). Although both isoforms are biologically active the relative importance of full length and mature IL-33 forms *in vivo* remains unclear (2, 21). In a mouse model of lung delivery of recombinant adenoviruses encoding IL-33 isoforms the full-length IL-33 induced inflammation in an ST2-independent fashion, but not pulmonary eosinophilia, goblet cell hyperplasia, or Th2 skewing, whereas mature IL-33 induced ST2-dependent Th2-associated effects. Both isoforms had similar effects on gene expression, suggesting that the different effects are due to differential utilization of the ST2 receptor (22). In addition, in a mouse model of DNA cancer vaccine, delivery of either full-length or mature IL-33 as an immunoadjuvant induced potent Th1 and cytotoxic T cell (CTL)-associated anti-tumor immunity and complete regression of established TC-1 tumor in mice. Interestingly, the full-length IL-33 was more potent than mature IL-33 in expanding the humoral immune response (23).

### The IL-33/ST2 axis

IL-33 exert its cytokine activity through binding to its primary specific receptor ST2, which is dependent on the co-receptor, IL-1 receptor accessory protein (IL-1RAcP), and the adaptor protein MyD88 for signaling (24). The crystal structure of IL-33 with ST2 has revealed that surface charge complementarity is crucial for specific binding (25). The gene that encodes for ST2 produces its transmembrane receptor but also produces a soluble form of ST2 (sST2), which acts as a binding decoy for IL-33 and thus down-modulates IL-33 activity during inflammatory responses, such as in experimental allergic asthma (26) and collagen-induced arthritis (27). Most hematopoietic cells express ST2. ILC2s, some Treg cells, and mast cells are the primary tissue-resident cells that constitutively express high levels of ST2, implying that these cells are initial targets of IL-33 (3). Non-hematopoietic cells, including endothelial cells, epithelial cells, and fibroblasts, are reported to express ST2 and respond to IL-33, although the *in vivo* consequences of signaling in these populations are less well-characterized.

**Figure 2:**
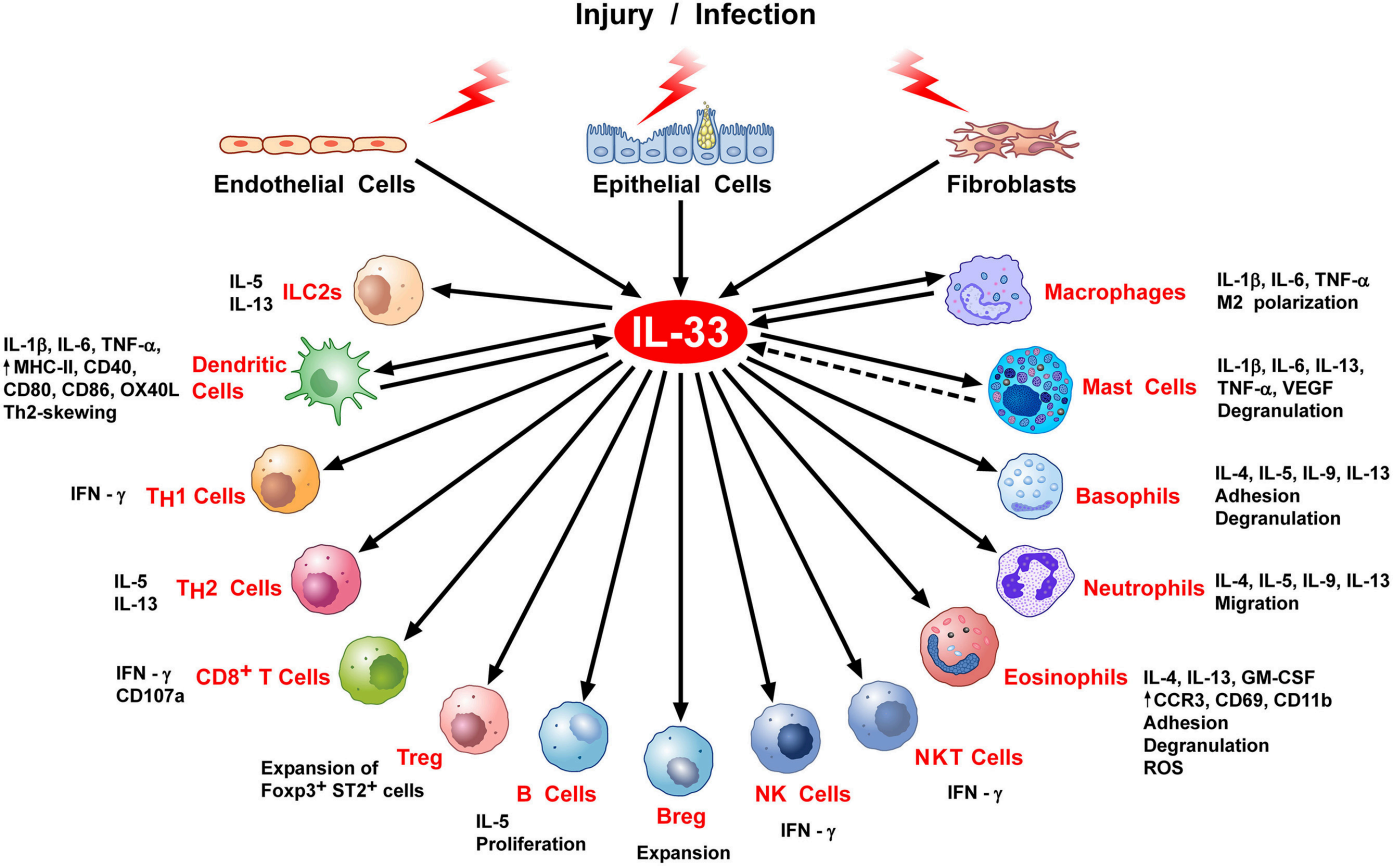
Stimulation of immune cells by IL-33. IL-33 is secreted by epithelial, endothelial, or fibroblasts in response to various stimuli, including infection or cell stress causing injury. IL-33 exerts its biological activities through binding to its specific receptor ST2 expressed by most hematopoietic cells. IL-33/ST2 can stimulate a variety of immune cell reactions, including: an atypical Th2-type of immune response through production of IL-5 and IL-13 by ILC2 and Th2 cells; IFNγ production by NK, NKT, and Th1 cells; CD107a exposure and IFNγ production by activated CD8 T cells; *in vivo* expansion of ST2^+^ Treg cells B and Breg cells; release of inflammatory cytokines (e.g., IL-1β, IL-6, TNFα) by DC, macrophages and mast cells; M2 macrophage polarization; degranulation of mast cells, basophils, and eosinophils; neutrophil migration; phenotypic, and functional activation of DC and eosinophils. There is some evidence that activated DCs, macrophages, and perhaps mast cells can produce IL-33.

In hematopoietic cells, IL-33 acts primarily on immune cells associated with type 2 and regulatory immune responses, including ILC2s, Th2 cells, eosinophils, mast cells, and basophils, as well as subsets of dendritic cells, myeloid-derived suppressor cells, and Tregs (28). However, it is now clear that the action of IL-33 is not limited to the activation of type-2 immune responses. Indeed, recent studies have revealed important roles of IL-33 in the activation of immune cells involved in type-1 immunity, such as Th1 cells, NK cells, CD8^+^ T cells, neutrophils, macrophages, B cells, and NKT cells (19, 29, 30). This pleiotropic nature of IL-33 (Figure 2) is likely to explain why IL-33 has been implicated in a wide variety of non-allergic diseases, including infectious diseases (fungal, helminth, protozoa, bacterial, and viral infection), cardiovascular diseases, chronic obstructive pulmonary disease (COPD), fibrotic diseases, musculoskeletal diseases, inflammatory bowel diseases, diseases of the central nervous system (Alzheimer), graft vs. host disease (GVHD), obesity, diabetes, and cancer (3).

## Immune cell targets of IL-33 affecting tumor-immune responses

### CD4^+^ Th cells

Naïve CD4^+^ T helper cells constitutively express ST2 and stimulation with IL-33 skews their differentiation toward a Th2 phenotype. CD4^+^ T cells are needed in the effector phase of a protective antitumor immune response against tumors lacking MHC class II (31). However, human CD4^+^ T cells can suppress tumors expressing adequate levels of MHC class II and self-antigens on their surface, through secretion of IFN-γ or direct tumor killing (32). Interestingly, Villareal et al. demonstrated that IL-33 can be an effective adjuvant when combined with an HPV16 E6/E7-encoded DNA vaccine, enhancing both antigen specific CD4^+^ and CD8^+^ IFN-γ^+^ T cells, and antigen specific IgG concentration in the serum, leading to regression of established TC-1 tumor in mice (23). In accordance with this study, Mousa Komai-Koma et al. showed that IL-33 may promote CD4^+^ T helper 1 (Th1) differentiation by a mechanism depending on IL-12 and ST2. IL-33 and IL-12 synergistically increase both ST2 and IL-12R expression in early activated CD4^+^ T cells. These data indicate that IL-33 promotes Th1 cell development, while it is ineffective on mature Th1 cells (33). A possible explanation for such differences is that ST2 expression is induced only in early-TCR activated naïve CD4^+^ T cells and is then gradually inhibited when Th1 cells fully mature. Although the signaling pathway by which IL-33 enhances Th1 polarization is still unknown, it is likely that IL-33 inducing Th1 or Th2response depends on the cytokine milieu, in particular the balance of IL-12 and IL-4 levels *in vivo* (33). Of note, IL-33 also promotes the differentiation of IL-9-producing Th cells (34) which exert potent antitumor immune responses *in vivo* (35, 36).

### CD4^+^ Treg cells

ST2/IL-33 signaling is known to expand suppressive CD4^+^ Foxp3^+^ GATA3^+^ Treg cells *in vivo* and *in vitro* (37). IL-33-expanded Tregs express ST2 and can be found in several immune and non-immune tissues exerting potent suppressor function in a variety of pathological conditions, such as autoimmunity, inflammation, transplantation, and allergy (38). ST2^+^ Treg expansion can be mediated by IL-33 signaling in DCs, through production of IL-2, which selectively expands ST2^+^ Tregs (39). In the intestine, particularly rich in ST2^+^ Treg cells, IL-33 signaling stimulates transforming growth factor (TGF)-β1-mediated differentiation of Treg cells and provides a signal for Treg-cell accumulation and maintenance in inflamed tissues (40). In Apc^Min/+^ mice, epithelial-derived IL-33 promoted the expansion of ST2^+^ Treg cells in the colon correlating with increased tumor burden (41, 42). A similar observation was recently reported in the CT26 adenocarcinoma model, where rIL-33 administration to tumor-bearing mice promoted, while IL-33 blockade reduced, the expansion of ST2^+^ Treg cells in tumor tissue and spleen (43). Moreover, IL-33 blockade reduced accumulation of Treg cells in tumor microenvironment and inhibited tumor growth in a preclinical model of human non-small-cell lung cancer (NSCLC) xenografts (44). In contrast, some studies have reported inhibitory effects of IL-33/ST2 on Treg cells expansion. In a melanoma mouse model, IL-33 was shown to inhibit Treg infiltration in the tumor microenvironment indirectly, through stimulation of MDSCs, which had reduced capacity to induce the differentiation or expansion of Treg cells *in vitro* (45). A recent study using reciprocal bone marrow chimeras in a mouse model of sporadic colon cancer, genetic ablation of ST2 in both hematopoietic and non-hematopoietic compartments leads to increased tumor-infiltrating ST2^+^ Foxp3^+^ Tregs and enhanced colon tumor development (46).

### CD8^+^ T cells

Unlike CD4^+^ T cells, only effector CD8^+^ T cells or polarized Tc1 cells, but not naïve and early activated CD8^+^ T cells, express ST2 (47). High expression of ST2 in CD8^+^ T cells cultured in Tc1 polarizing conditions is regulated by T-bet, a master transcription regulator of Th1 effector functions. Moreover, it was shown that IL-12 and IL-33 synergistically increased T-bet and Blimp1, transcription factors critical for effector fate of CD8^+^ T cell (47). Recent studies from transplantable solid tumor models have indicated a direct role of exogenous IL-33 in promoting antitumor CD8^+^ T cell immunity using either IL-33 transgenic mice (48), IL-33 DNA as vaccine adjuvant (23), or IL-33 expressing tumor cells (49). Systemic administration of rIL-33 in melanoma tumor bearing mice, promoted expansion, increased tumor infiltration and effector function of antigen-specific CD8^+^ IFN-γ^+^ T cells by both a direct or DCs-mediated effect (50). In the aggressive C1498 acute myeloid leukemia (AML) model, IL-33 treatment significantly increased the percentage of effector memory liver CD8^+^ T cells leading to delayed leukemia development and improved overall survival (51). This finding suggest a role of exogenous IL-33 in promoting rapid expansion of the effector memory CD8^+^ T cell pool, consistent with the results from solid tumor models (23, 48). In this study the increased CD8^+^ T cells activation level up-regulates PD-1/PD-L1 expression *in vivo*, therefore combination of PD-1 blockade and IL-33 treatment further improves survival of leukemia-bearing mice (51).

### Type-2 innate lymphoid cells

Innate lymphoid cells (ILCs), belonging to the family of innate cells, are characterized by classic lymphoid cell morphology, but lack lineage-specific markers and somatically rearranged antigen receptors. Based on the expression of transcription factors, phenotypic markers, and effector cytokine production profiles, ILCs have been divided into three distinct subclasses: group 1 ILCs, group 2 ILCs, and group 3 ILCs (52). ILC are derived from a common lymphoid progenitor and possess a wide range of cell surface markers, many of which have only recently been elucidated (53). ILC2, originally identified in the mouse and human mesenteric lymph nodes as lineage marker negative, c-kit^+^, Sca-1^+^, IL-7Ra^+^, and ST2^+^ cells (54), were also found in lung, skin, and gut, while only a small number of circulating ILC2s can be detected in blood (55). They are involved in tissue repair (56), anti-helminth immunity (57), and allergic inflammation (58).These cells are dependent on transcription factor GATA-binding protein 3 for their development and maintenance (59). Activation of ILC2s by alarmins (IL-25, IL-33, and TSLP) secreted by epithelial cells upon cellular stress and tissue damage (55, 60), produce IL-5, IL-13 (54), IL-4, IL-6, IL-9, and amphiregulin which induce Th2 differentiation (61). This group of innate cells was often observed to infiltrate tumors in humans, but their role seems more frequently associated with cancer progression than restriction. Clinical studies suggested that increased numbers of ILC2s in peripheral blood of patients with gastric cancer, could contribute by cytokines they secrete to the immunosuppressive environment maintained by CD4^+^ T helper 2 (Th2), myeloid-derived suppressor cells (MDSC), and macrophages (62). ILC2s might also induce immune suppression *via* secretion of amphiregulin (63), which enhances Treg activity *in vivo* and can thereby inhibit antitumor immune responses induced by DC vaccination (64). The anti-tumoral activity of ILC2 was described for the first time by Ikutani et al. in a mouse model of lung metastatic melanoma. Following tumor induction, administration of rIL-33 induced the development of IL-5-producing ILC2, which recruited and maintained eosinophils responsible for tumor cell death and tumor metastasis prevention (65). Overexpression of IL-33 in several tumor cell lines induced high numbers of ILC2s, when transplanted in mice, with potent anti-tumoral activity. The latter study suggests that local production of IL-33 induces ILC2 to release CXCR2 ligands able to sustain the expression of CXCR2 on tumor cells and induce their apoptosis (66).

### NK and NKT cells

IL-33 directly activates both human (19) and mouse (30) NKT and NK cells inducing IFN-γ production via cooperation with IL-12, thus contributing to establish Th-1 immunity. During viral infection, IL-33/ST2 axis amplifies the expansion of NK cells and enhances host defense (67, 68). The role of IL-33 on NKT in cancer immunity is unknown. In contrast, a number of reports have analyzed the effects of IL-33 on NK cell expansion and/or activation in tumor-bearing mice. In mouse experimental metastasis models of B16 melanoma and Lewis lung carcinoma, transgenic expression of IL-33 in the host promoted the recruitment of cytotoxic NK cells to the pulmonary site that inhibited metastasis formation (48). *In vitro*, IL-33 directly activated NK cell cytotoxicity, stimulated NF-κB and up-regulated CD69 expression (48). Furthermore, B16 and 4T1 tumor cells overexpressing IL-33 implanted into syngeneic mice induced IFN-γ^+^ NK cells in tumor tissue that mediated IL-33 anti-tumoral effect (49). Similarly, increased frequencies of CD107a^+^IFN-γ^+^ NK cells were observed following exogenous administration of IL-33 in spleens and tumors of B16 melanoma-bearing mice (69). In contrast with these reports, previous studies in 4T1 breast cancer model showed that IL-33/ST2 signaling impairs NK cell activation. ST2-deficient mice bearing 4T1 tumors exhibited increased numbers of activated NK cells (IFN-γ^+^ CD27^high^ CD11b^high^, CD69^+^ KLRG^−^) and NK cytotoxic activity, with respect to wild-type (WT) counterparts. *In vivo* depletion of NK cells accelerated 4T1 tumor growth in ST2^−/−^ mice (70). Moreover, exogenous administration of IL-33 to WT 4T1 tumor-bearing mice decreased NK cell activation and cytotoxicity and promoted tumor progression (71), thus suggesting a detrimental role for IL-33 in NK cell-dependent anti-tumor responses. These contrasting results suggest that IL-33 may exert opposing effects on NK cells within the tumor microenvironment depending on the levels of IL-33 expressed and on the primary target cells.

### Macrophages

Several lines of evidence indicate that IL-33 amplifies the expression of M2 markers on macrophages *in vitro* and *in vivo*, thus promoting the suppressor function of tumor-associated macrophages (TAMs). Blockade of IL-33 abrogates the polarization of TAMs into (alternatively activated) M2 macrophages in a model of human non-small-cell lung cancer (44). Expression of IL-33 was found to stimulate the recruitment of M2-like macrophages into the cancer microenvironment in mouse models of colon (41, 72–74) and breast cancer (71) correlating with tumor progression. Of note, IL-33 stimulated macrophages to produce prostaglandin E_2_, which supported colon cancer stemness (73). Furthermore, in mouse tumor xenografts IL-33 was shown to promote metastasis through recruitment of M2-like TAMs (75). Recently, it was shown in the mouse monocyte/macrophage line RAW264.7 that IL-33 directly induces MMP-9 expression, which facilitates tumor progression, invasion, and angiogenesis (76). These observations indicate that IL-33/ST2 signaling on macrophages promotes M2 polarization, immunosuppression, and tumor progression.

### Dendritic cells

Although dendritic cells (DCs) express low levels of ST2 on their cell surfaces, they respond to IL-33 by up-regulating MHC-II, CD40, CD80, CD86, OX40L, CCR7, and by increasing production of several cytokines (IL-4, IL-5, IL-13, TNF-α, and IL-1β) and chemokines (CCL17 and CCL22) (77–80). In addition, IL-33-activated DCs promote an atypical Th2-type of immune response inducing IL-5- and IL-13-producing CD4^+^ T cells *in vitro* and *in vivo* (77, 79), which can be further amplified during allergic inflammatory response *via* ST2 (78, 81). *In vivo*, IL-33 exposure induces DC recruitment and activation in the lung (78, 79). IL-33 promotes the expansion of DCs from bone marrow (BM), by stimulating the secretion of basophils-derived GM-CSF. However, such IL-33 differentiated BM-DCs expressed low levels of MHC-II but high PD-L1 and PD-L2 immune checkpoints on the surface, and displayed reduced capacity to prime naïve T cells (82). In support of this potential tolerogenic effect, IL-33 has been shown to promote IL-2 secretion by murine DCs, thus supporting the *in vitro* and *in vivo* expansion of ST2-expressing Treg cells (39). In mice bearing 4T1 breast cancer IL-33 administration increased the percentage of splenic CD11c^+^ DCs expressing IL-10 (71). In contrast, in a murine AML model, systemic IL-33 administration promoted DC activation and “licensing” for cross-priming of tumor-reactive CD8^+^ T cells (51). Likewise, in EG7 lymphoma, B16, and inducible Braf^V600E^PTEN melanoma models exogenous IL-33 activated myeloid DCs within the tumor microenvironment increasing antigen cross-presentation and restoring anti-tumor T cell activity in a ST2, MyD88, and STAT1-dependent manner (50). On the whole, these results suggest that IL-33 depending on the context can stimulate DC antigen presentation and, thus, anti-tumor immune responses, or induce tolerogenic features, thus supporting tumor growth.

### Myeloid-derived suppressor cells

Myeloid-derived suppressor cells (MDSCs) are closely related to granulocytes and monocytes but differ from them in that they are absent in healthy individuals but expand under pathological conditions, such as cancer, exerting potent immune suppressive role (83). Several reports described the ability of IL-33 to expand MDSCs *in vivo* during tumorigenesis. In 4T1 breast cancer model, IL-33 has been reported to promote MDSC expansion (71). Exogenous administration of IL-33 increased intratumoral and systemic accumulation of CD11b^+^ Gr-1^+^ MDSCs that expressed TGF-β1 and IL-13α1R with higher incidence of monocytic vs. granulocytic MDSCs. Fittingly, absence of IL-33/ST2 signaling reduced the accumulation, proliferation, and immunosuppressive ability of MDSCs in tumor-bearing mice (71, 84). Moreover, IL-33 upregulated in MDSCs the expression and activity of arginase-1 *in vitro* and activated NF-κB and MAPK signaling *in vivo*, which augment their immunosuppressive ability (84). Conversely, *in vitro* IL-33 was shown to negatively regulate MDSC development from BM progenitor cells inhibiting the differentiation of granulocytic MDSCs (G-MDSCs), but not of monocytic MDSCs (M-MDSCs). In addition, IL-33-treated BM-derived MDSCs exhibited diminished immunosuppressive capacity, reduced inhibition on T-cell proliferation and IFN-γ production, and diminished production of ROS (45). In B16 melanoma mouse models, IL-33 administration was shown to decrease MDSCs accumulation in the spleen and tumor microenvironment (45, 69). These evidences indicate that IL-33 may promote or halt MDSCs expansion depending on the tumor type.

### Neutrophils

IL-33 directly acts on murine neutrophils in the lungs (85). Indeed, IL-33-treated neutrophils produced IL-4, IL-5, IL-9, and IL-13 and displayed a distinct gene expression profile in contrast to resting and lipopolysaccharide (LPS)-treated neutrophils. These neutrophils were found in the lungs of ovalbumin (OVA)-induced mouse model of asthma. Adoptive transfer of IL-33-driven neutrophils significantly worsened the severity of the disease in this model (86). *In vitro*, IL-33 promoted IL-4, IL-5, IL-9, and IL-13 expression in murine neutrophils in time- and dose- dependent manner. IL-33-induced neutrophils expressed high levels of CXCR1, CCR1, IL-1R2, and CXCR2 mRNAs compared with LPS-induced neutrophils. In a mouse model of choriomeningitis virus-induced hepatitis, IL-33 promoted neutrophil recruitment in the liver and dampened liver injury by limiting T-cell activation. Liver neutrophils displayed an immunosuppressive phenotype, characterized by high levels of arginase-1, iNOS and IL-10 (87). Thus, IL-33 may promote inflammatory or regulatory neutrophils, depending on the pathological condition. Little is known about the effects of IL-33 on neutrophils in tumor immunity. In a mouse model of ectopic CT26 colon carcinoma, systemic chemotherapy with irinotecan induced intestinal mucositis, associated with the induction of IL-33, and increased neutrophil accumulation in the intestine. Supernatants from intestine explants treated with irinotecan enhanced migration of neutrophils *in vitro* in an IL-33/CXCL1/2/CXCR2-dependent manner. Importantly, IL-33 blockade reduced mucositis and enabled prolonged irinotecan treatment of ectopic colon carcinoma leading to a beneficial outcome of the chemotherapy. These results suggest that inhibition of the IL-33/ST2 pathway may represent a novel approach to limit mucositis and improve the effectiveness of chemotherapy (88).

### Eosinophils

Administration of IL-33 in mice causes massive tissue infiltration of eosinophils and elevations of typical type 2 cytokines such as IL-5, IL-9, and IL-13, contributing to allergy and fibrosis (89). These responses also occurred in *RAG* knockout mice, suggesting that innate cells, particularly IL-5 producing ILC2s, were the direct target of IL-33 (54). However, IL-33 is now known to act directly on eosinophils leading to upregulation of CCR3, CD69, and CD11b, production of chemokines (CCL17, CXCL2, CXCL3, and CXCL10), and cytokines (IL-6, IL-13, GM-CSF) (90). IL-33 sustained eosinophil survival *in vivo* in a ST2-dependent manner and *via* autocrinous production of GM-CSF. Moreover, IL-33 and GM-CSF promoted the production of IL-4 and IL-13 by eosinophils, which in turn favored macrophage polarization toward M2 phenotype (91). In a murine model of melanoma, *in vivo* expansion of innate IL-5–producing ILC2 cells following systemic IL-33 injection played an important role for eosinophil recruitment and metastasis control. Innate IL-5-producing cells were increased in response to tumor invasion, and their regulation of eosinophils is critical to halt tumor metastasis (65). We reported that in transplantable B16 melanoma models, IL-33 restricted tumor growth and inhibited lung metastasis through recruitment and activation of eosinophils. Indeed, ST2-deficient mice presented an increased metastatic load and reduced lung eosinophilia compared to wild type mice. Depletion of eosinophils completely abolished the anti-tumor effects of IL-33 administration (69). Kim et al. also reported substantial expansion of intratumoral eosinophils in mice transplanted with IL-33-expressing tumor cells (EL4, CT26, and B16); however, their role in IL-33-induced anti-tumor effects was not addressed (66). In our study, IL-33 expanded eosinophils expressed T cell-attracting chemokines and induced NK and CD8^+^ T cell-recruitment at the subcutaneous tumor site, but not at the lung metastatic site. In addition, IL-33 activated eosinophils *in vitro* enhancing CD11b, CD69, and granzyme B expression and activating cytotoxic functions against melanoma cells (69). These findings suggest that depending on the tumor site, eosinophils may play an accessory role, supporting the recruitment of tumor-reactive CD8^+^ T cells (92), or a direct cytotoxic effect against tumor cells. Indeed, IL-33 can potently activate human eosinophils, enhancing adhesion, promoting survival, and inducing ROS production and degranulation (93, 94). Of note, degranulation of eosinophils has been shown *in vivo* in proximity of tumors (95, 96) and after *in vitro* stimulation, resulting in efficient killing of target mouse (97) and human (98) tumor cells, highlighting the tumoricidal properties of eosinophils (99).

### Mast cells

IL-33 activates its receptor complex (ST2: IL-1RAcP) on human mast cells (100) and basophils (19). IL-33 synergizes with IgE- and non-IgE-dependent stimuli to release cytokines from human mast cells (101). Similarly, IL-33 augments substance P-induced vascular endothelial growth factor (VEGF) production from human mast cell lines (102). The latter findings are clinically relevant because VEGFs produced by human mast cells (103) and basophils (104) play an important role in chronic inflammation and in tumor growth (105). We have demonstrated that IL-33 up-regulated the Fcγ receptor type IIa and synergistically enhanced immune complex-triggered activation of human mast cells (106). Collectively, these findings demonstrate that IL-33 can synergistically potentiate the immunologic and non-immunologic release of mediators from human and rodent mast cells. Single cell analysis demonstrated that IL-33 increased both the number of degranulating and chemokine-producing mast cells and the magnitude of individual mast cell response (107). The relevance of IL-33-mediated mast cell response has been found also *in vivo* in several pathological conditions, including cancer. Mast cells and basophils are known to infiltrate several types of tumors but, due to the wide range of mediators they release, it is difficult to define their specific pro- or anti-tumoral activity (103). Mast cell activation by IL-33 may occur in a number of tumor types. In skin cancers, mast cells accumulated with IL-33 expressing fibroblasts in UV-exposed murine skin samples (108). In the *Apc*^*Min*/+^ mouse model, IL-33 deficiency reduced tumor burden (109, 110) and decreased mast cell density in polyps as well as suppressed the gene expression of mast cell-derived proteases and cytokines that promote angiogenesis, Treg function, and MDSC recruitment within the tumor microenvironment (111–113).

### Basophils

Human basophils constitutively express ST2 which is induced by IL-3 (114, 115). Although IL-33 alone failed to directly induce degranulation of human basophils, it exerted priming effects. It enhanced degranulation and IL-4 production in response to IgE cross-linking (114). By contrast, IL-33 alone activated unprimed murine basophils *in vitro* (116). Recently, Rivellese and collaborators, using highly purified human basophils, elegantly demonstrated that IL-33 alone induces the release of low but detectable amounts of IL-4 (117). IL-33 synergistically potentiated IL-4 production induced by IL-3 or anti-IgE. Interestingly, IL-33 did not induce basophil degranulation, as evaluated by the membrane expression of CD63, but significantly enhanced IgE-mediated histamine release. Collectively, these findings indicate that IL-33 can activate human basophils presumably through the engagement of ST2. Furthermore, IL-33 can enhance IL-3- and anti-IgE-mediated basophil degranulation, histamine secretion, and cytokine production. The role of basophils in anti-cancer immunity is poorly characterized and the effects of IL-33 in stimulating or regulating basophils responses in cancer are unknown. In mouse models of melanoma, Sektioglu et al. reported that intratumoral basophils enhanced CD8^+^ T cell infiltration *via* production of the chemokines CCL3 and CCL4, contributing to tumor rejection following Treg cell depletion (118). Recently, low circulating eosinophils and basophils were associated with poor prognosis in CRC patients (119). Moreover, basophils in tumor-draining lymph nodes of both mice and pancreatic ductal adenocarcinoma patients correlated with Th2 responses in tumors and poor prognosis (120).

## Role of IL-33 in cancer: evidences from experimental tumor models

Accumulating evidences indicate that the axis IL-33/ST2 plays a role in tumor immunity. However, both pro-tumoral and anti-tumoral functions have been reported and the current literature suggest that IL-33 may differently affect tumor immunity depending on the tumor type, immune cell targets and on cooperating microenvironmental factors (121).

### Breast cancer

The majority of reports point to a pro-tumoral role of IL-33 in breast cancer models. In mice, the IL-33/ST2 axis was shown to promote the growth and lung and liver metastases of 4T1 mammary tumors facilitating the intratumoral accumulation of immunosuppressive myeloid (Gr-1^+^ TGF-β1^+^ MDSC, IL-10-expressing CD11c^+^ DCs, and alternatively activated M2 macrophages), ILC2, and Treg cells, while dampening the expansion of activated NK cells (70, 71, 84). Lukic group demonstrated that IL-33/ST2 axis promotes the expression of pro-angiogenic VEGF in tumor cells and attenuates tumor necrosis, thus facilitating mammary tumor growth (122). In contrast, another study reported that transgenic overexpression of IL-33 in 4T1 breast cancer cells reduces tumor growth and metastasis *in vivo* (49). *In vitro*, IL-33 acts as a critical tumor promoter during epithelial cell transformation and sustains breast cancer tumorigenesis (123). Recently, it has been reported that IL-33 overexpression in human ER-positive breast cancer cells results in resistance to tamoxifen-induced tumor growth inhibition, by promoting cancer stem cell properties (124). These findings indicate that in breast cancer IL-33/ST2 exert both intrinsic and extrinsic pro-tumoral function favoring tumorigenesis and stemness and reducing anti-tumor immunity.

### Colorectal cancer

A number of evidences from mouse models point to an important role of the IL-33/ST2 axis in promoting colorectal cancer (CRC) tumorigenesis, progression and malignancy (125). In the Apc^Min/+^ mouse model for human familial adenomatous polyposis, abrogation of the IL-33/ST2 axis by knockout of IL-33 (109) or ST2 (41, 110) inhibits proliferation, induces apoptosis, and suppresses angiogenesis, thus decreasing tumor number and size. Accordingly, overexpression of IL-33 in MC-38 mouse CRC cells results in increased *in vitro* proliferation and enhanced tumor growth and liver metastasis after orthotopic transplant in syngeneic mice through tumor-derived IL-33-induced recruitment of CD11b^+^ GR1^+^ and CD11b^+^ F4/80^+^ myeloid cells and angiogenesis (126). In addition, expression of IL-33 in human CRC cells promoted their growth and metastasis *in vivo* and reduced the survival of recipient nude mice (127). Likewise, IL-33/ST2 signaling in CRC tissues promoted the malignant growth and metastatic spread of CRC through modification of the tumor microenvironment (72). In a recent study, it was shown that IL-33 overexpression or exogenous administration of IL-33 to human or murine colon cancer cells enhanced cell growth *in vivo* and promoted colon cancer cell stemness through an immune-associated mechanism (73). In contrast, some studies have reported an anti-tumoral function of IL-33 in CRC models. Abrogation of ST2 signaling in CT26 mouse adenocarcinoma cells enhanced tumor development after subcutaneous transplant into syngeneic BALB/c mice (74). In the azoxymethane (AOM)/dextran sodium sulfate (DSS) model, IL-33-deficient mice were shown to be highly susceptible to cancer-associated colitis showing increased tumor number, size, and grade, due to a protective function of IL-33 in regulating an IgA-microbiota axis in the intestine (128). Fittingly, in a mouse model of sporadic colon cancer tumorigenesis in the absence of preexisting inflammation lack of IL-33 signaling enhanced colon tumorigenesis, while IL-33 treatment reduced tumor growth in the transplantable MC38 model, via IFN-γ-mediated antitumor immune response (46). These evidences suggest that in the absence of preexisting chronic or acute inflammation the homeostatic release of IL-33 by dying colon epithelial cells protects against the initiation and development of sporadic colon cancer.

### Lung cancer

The IL-33/ST2 axis plays a critical role in various inflammatory lung diseases, including asthma and fibrosis (129). However, few studies have investigated the contribution of this pathway to lung cancer. Akimoto et al. reported that ST2 expression in human and murine lung cancer is inversely correlated with metastatic potential (130). Exposure to IL-33 enhanced oncotic cell death of ST2^+^ low-metastatic Lewis lung carcinoma cells, but not of ST2^−^ high-metastatic cells, thus suggesting that IL-33 enhances lung cancer progression by selecting for malignant cells. Moreover, *in vitro* stimulation with IL-33 promoted the migration and invasiveness of human lung A549 cells (131) and enhanced the growth and metastasis of primary non-small-cell lung cancer (NSCLC) cells after xenotransplant into immunodeficient mice (132). *Vice versa*, IL-33 blockade efficiently inhibited tumor growth of patient-derived NSCLC xenografts, abrogating M2 polarization of macrophages, and reducing the accumulation of Treg cells within the tumor microenvironment (44). In mouse lung tumor models stable transfection of *IL-33* gene into tumor cells inhibited tumor growth and metastatic spread *in vivo* (49, 133). Of note, IL-33 expressing metastatic A9 lung cancer cells, a TC1-derived cell line with spontaneous down-regulation of MHC-I, restored MHC-I expression and immune recognition in mice (133). Although these evidences indicate that IL-33/ST2 expression in lung tissue or cancer cells fuels tumor growth, the contribution of IL-33 to anti-tumor adaptive immune responses against this cancer remains to be established.

### Melanoma

Accumulating evidences indicate that in melanoma models IL-33 exert anti-tumoral and anti-metastatic effects by conditioning the local immune environment. Systemic injections of IL-33 significantly inhibit tumor growth in mice bearing subcutaneous B16.F10 melanomas (50, 69) and in BRAF^V600E^PTEN-inducible melanoma model (50). Dominguez et al. reported that IL-33 both directly stimulated CD8^+^ T cell expansion and IFN-γ production and activated myeloid dendritic cells (mDCs) increasing antigen cross-presentation. Of note, combination therapy with rIL-33 and agonistic anti-CD40 antibodies demonstrated synergistic anti-tumoral activity in this model (50). Using subcutaneous and experimental metastasis B16 melanoma models, we recently demonstrated that IL-33 inhibits melanoma tumor growth and pulmonary metastasis *in vivo* in an eosinophil-dependent manner (69). In a previous study, it was shown that IL-33 transgenic mice inhibit tumor metastasis in the B16 melanoma model (48). Likewise, transgenic expression of a secretable mature form of IL-33 by plasmid DNA in B16 tumor cells reduced tumor growth and metastasis *in vivo* (49). In these models, both NK and CD8^+^ T cells were required for the anti-tumoral effect of IL-33. Instead, Kim et al. showed that subcutaneous injection of adenoviral vector-transfected B16 melanoma cells engineered to secrete IL-33 did not develop palpable tumors in mice, in a CD8^+^ T- and NK-independent manner, through expansion of CXCR2 ligands-secreting intratumoral ILC2 (66). *Ex vivo* explanted IL-33-expressing B16 tumors exhibited higher reactive oxygen species (ROS) levels and increased CXCR2 expression and apoptosis thus suggesting a role for IL-33 in creating a hypoxic tumor microenvironment supporting ILC2-induced apoptosis (66). The explanation for such diverse immune-mediated anti-tumor mechanisms needs further investigations, although it is plausible that different local concentrations of IL-33 within the tumor microenvironment may stimulate different responses.

### Other tumors

IL-33 has been reported to exert an anti-tumoral role in other tumor models. In a human papilloma virus (HPV)-associated model for cancer immunotherapy IL-33 was shown to act as a potent vaccine adjuvant augmenting Th1 and CD8^+^ T-cell responses, inducing anti-tumor immunity *in vivo* (23). Enforced expression of IL-33 in a large collection of murine tumor cell lines, including CT26 colon carcinoma, EL4 lymphoma, B16 melanoma, Lewis lung carcinoma (LLC), A9, and 4T1 lung cancer, results in reduced tumor growth *in vivo* (49, 66, 133). In a murine acute myeloid leukemia (AML) model administration of IL-33 significantly inhibited leukemia growth and improved mice survival rate in a CD8^+^ T cell dependent manner (51). Notably, combination of PD-1 checkpoint blockade with IL-33 further prolonged mice survival, and induced complete leukemia regression in 50% of animals. The latter report represents the first evidence for combining IL-33 with immunotherapy targeting immune checkpoint inhibitors. Recently, administration of rhIL-33 was shown to expand Vγ9 T cells improving the therapeutic response to phosphoantigen in preventing tumor growth in a humanized mouse lymphoma model (134). Pro-tumoral effects were observed in several other types of experimental cancers (135). In an organotypic culture model, carcinoma-associated fibroblasts (CAFs) were found to express high levels of IL-33 which promoted cancer invasive behavior of head and neck squamous cell carcinoma (HNSCC) cells (136). In gastric cancer, IL-33 exerted a pro-tumorigenic function inducing cancer cell invasion by stimulating the secretion of MMP-3 and IL-6 via ST2-ERK1/2 pathway (137) and activation of the JNK pathway (138) conferring chemotherapy resistance *in vitro*. Finally, in mouse models of cholangiocarcinoma (CCA), a malignant neoplasm of the biliary-duct system, administration of IL-33 was found to increase biliary tumorigenesis through an increase of IL-6 expression in tumor tissue (139) and to enhance cholangiocyte proliferation, by increasing the numbers of IL-13-producing ILC2s (140). The effects of IL-33 in experimental cancers are summarized in Table 1.

**Table 1:** Role of IL-33 in experimental tumors.

**Role**	**Cancer type**	**Species**	**Reported effects**	**References**
Pro-tumoral	Breast	Mouse	IL-33/ST2 favors accumulation of immunosuppressive myeloid cells, Tregs, and ILCs. Increases tumor pro-angiogenic VEGF.	(70, 71, 84, 122)
		Human	IL-33/ST2 promotes epithelial cell transformation and breast tumorigenesis. Confers breast cancer endocrine resistance and cancer stem cell properties.	(123, 124)
	CRC	Mouse	IL-33/ST2 increases polyposis in Apc^Min/+^ mice. Enhances tumor proliferation, growth, and angiogenesis. Promotes accumulaion of immunosuppressive myeloid cells.	(41, 72, 73, 109, 110, 126)
		Human	IL-33/ST2 expression increases tumor growth and metastasis in nude mice, tumor recruitment of prostaglandin E_2_-producing macrophages, and M2 polarization. Promotes colon cancer cell stemness.	(72, 73, 127)
	Lung (NSCLC)	Human	ST2 increases tumor invasiveness and metastatic potential by selection of malignant cells. IL-33 blockade suppresses tumor growth and metastasis in nude mice, M2 polarization, and Treg accumulation.	(44, 130–132)
	Gastric	Human	IL-33 induces cancer cell invasion and confers chemotherapy resistance *in vitro*.	(137, 138)
	HNSCC	Human	IL-33 expression in CAFs promotes cancer invasive behavior.	(136)
	CCA	Mouse	IL-33 increases biliary tumorigenesis in mice via IL-6. It induces cholangiocyte proliferation mediated by ILC2s.	(139, 140)
Anti-tumoral	Melanoma	Mouse	Transgenic host or tumoral expression or IL-33 administration inhibits tumor growth and pulmonary metastasis, via stimulation of NK, CD8^+^ T cells, DCs, ILC2s, and eosinophils.	(48–50, 66, 69)
	Lung	Mouse	IL-33 expression in lung cancer cells reduces tumor growth *in vivo*, restores MHC-I expression and immunovisibility.	(49, 133)
	CRC	Mouse	IL-33/ST2 signaling negatively correlates with tumor progression of CT26 and MC38 tumors. IL-33 induced IFNγ-mediated antitumor immune response. In a model of DSS-induced colitis IL-33 exerts a protective function regulating in testinal IgA-microbiota axis.	(46, 74, 128)
	AML	Mouse	IL-33 inhibits leukemia growth, improves mice survival rate through CD8^+^ T cells and synergizes with PD-1 blockade to promote tumor rejection.	(51)
	Lymphoma	Mouse	IL-33 expands anti-lymphoma Vγ9 T cells and improves the therapeutic response to phosphoantigen.	(134)

*CRC, colorectal cancer; NSCLC, non-small cell lung cancer; HNSCC, head and neck squamous cell carcinoma; CCA, cholangiocarcinoma; AML, acute myeloid leukemia; CAF, cancer associated fibroblasts*.

## IL-33/ST2 as a biomarker predictive of cancer progression and patients survival

The recent literature regarding IL-33 involvement in tumorigenesis is controversial, since IL-33 seems to have dual, pro-inflammatory or protective, roles depending on the cellular and cytokine context. Some studies have shown a positive correlation between IL-33 expression in tumor tissue and a favorable prognosis in cancer patients. For example expression levels of IL-33 and ST2 were significantly down-regulated in both adenocarcinoma and squamous cell carcinoma of lung tissues when compared to adjacent normal lung controls (141). Furthermore, plasma IL-33 levels were elevated during the early stage of lung cancer and decreased with advanced cancer stages, probably due to lung volume reduction containing bronchial epithelium and vascular endothelium as sources of IL-33 (142). The observed decreases indicate that the expression levels of IL-33 are inversely associated with lung cancer progression (143). Of interest, in hepatocellular carcinoma resected tissues expression of IL-33 by intratumoral effector memory CD62L^−^KLRG1^+^CD107a^+^ CD8^+^ T cells was shown to be a prognostic marker for increased patients survival (144). Serum levels of IL-33 were significantly higher in patients with breast cancer compared to patients with benign breast diseases, so the local expression of IL-33 may be a marker for differentiating malignant from normal/benign tissues (145, 146). IL-33 expression in adjacent tissues also tends to be higher compared to normal tissues, suggesting that adjacent non-cancerous tissues may be similarly relevant to cancers in terms of anti-tumor immunity. Local IL-33 expression may also increase intratumoral accumulation of immunosuppressive lymphoid cells in patients with breast cancer (145, 146). Plasma sST2 levels and nuclear IL-33 expression were found to be increased in endothelial cells in bone marrow biopsies from patients with myeloproliferative neoplasms, whereas low/undetectable levels were found in healthy donors (147). It has been proposed that IL-33 induces the production of cytokines and growth factors that promote myelopoiesis and facilitates the development of leukemia by inducing and/or enhancing the proliferation of hematopoietic progenitors in the bone marrow microenvironment from patients with chronic myeloid leukemia (147).

In other clinical conditions, increased IL-33 expression was inversely correlated with the overall survival of cancer patients. Serum IL-33 levels were increased in renal cell carcinoma (RCC) patients compared to healthy volunteers (148). Such over-expression was associated with advanced tumor-lymph node-metastasis (TNM) stage, resulting in reduced survival and increased risk of recurrence in patients. Mechanistically, it has been shown that IL-33 enhances RCC cell growth *in vivo* and prevents chemotherapy-induced tumor apoptosis *in vitr*o via JNK signaling activation in tumor cells (149). Similarly, both IL-33 and ST2 were up-regulated in ovarian tumors compared to normal ovary and ovarian benign tumors, and the expression levels were further increased in tumor tissues at the metastatic site (150). It has been proposed that IL-33/ST2 axis promotes ovarian cancer migration and metastasis through regulation of ERK and JNK signaling pathways (151). Furthermore, most head and neck squamous cell carcinoma (HNSCC) cases with a low invasion pattern grading score (IPGS) showed low or no expression of IL-33, whereas most HNSCC cases with high IPGS displayed abundant expression of IL-33 in CAFs and in cancer cells. This observation suggests a paracrine effect of IL-33 as a result of the crosstalk between tumor cells and surrounding stromal cells. Hence, CAFs overexpressing IL-33 promote the induction of epithelial-to-mesenchymal trans-differentiation, eventually leading to tumor progression and poor prognosis (136). Higher IL-33 expression was described in glioma tissue compared to normal brain tissues at both transcriptional and translational levels (152, 153). It has been proposed that IL-33 stimulates cell migration through the expression of matrix metalloproteinases (MMP2/MMP9) *via* the ST2/ NF-κB pathway, thereby promoting cell invasion and tumor growth (154). Higher expression of IL-33 and total ST2 (ST2L and sST2) have also been reported in colorectal cancer (CRC) tissues compared to adjacent normal tissues (127). This observation could be explained considering that inflammatory cytokines are important components of the CRC microenvironment and colon cancer progression is closely related to chronic inflammation. Increased IL-33 expression is observed in poorly differentiated human CRC cells, which is associated with poor survival in patients with metastatic colon cancer (73).

The relation between IL-33 and sST2 serum levels and the survival of patients suffering from liver cirrhosis (LC) and hepatocellular carcinoma (HCC) has not been determined yet. No significant difference in IL-33 serum levels was found in HCC compared to LC. IL-33 levels did not correlate with overall survival or liver function parameters, whereas sST2 levels were significantly elevated in LC and HCC patients, compared to healthy subjects, and were associated with overall survival of HCC. Therefore, its function remains to be clarified (155). Similarly, IL-33 protein levels were significantly lower in gastric cancer tissues than adjacent tissues. These levels were associated to the depth of tumor invasion and the morphology of the tumor, suggesting that IL-33 is involved in the process of inflammatory reaction in the development of gastric cancer, while it is not significantly associated with the overall survival of these patients (156).

## Analysis of IL-33 mutations and isoforms in cancer

**Figure 3:**
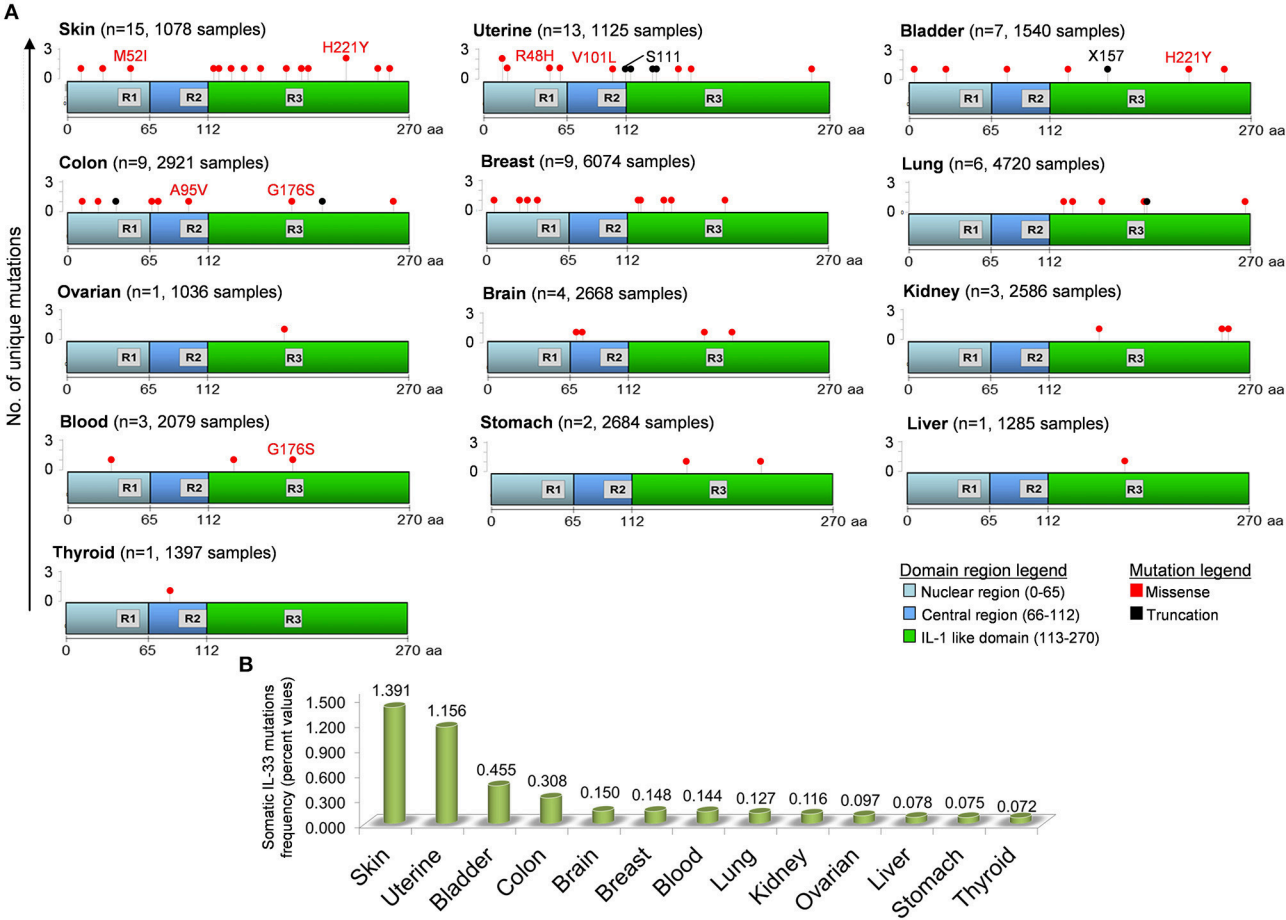
Distribution of unique somatic IL-33 gene mutations in tumors and in the domain regions of the IL-33 protein. **(A)** IL-33 gene mutations in each tumor have been obtained by inferring the publicly available cBioPortal database for analysis of large-scale cancer genomics data sets (http://www.cbioportal.org/). For each tumor type the number of mutations (*n*) and the total number of sample patients is depicted. The IL-33 gene mutation positions are aligned to the three principal regions of the IL-33 protein. R1, Chromatin binding domain (M45XLRSGXXI53); R2, Cleavage site for inflammatory Proteases (T90VECFAFGISGVQKYTRALHDSS112); R3, Cleavage site for caspase 3 and caspase 7 (D175GVDG179). Some missense (red text) and truncation (black text) mutations are depicted. **(B)** Somatic IL-33 gene mutation frequencies calculated by data analyzed in **(A)** and indicated for each tumor type.

IL-33 gene undergoes a certain number of somatic mutations during tumorigenesis. Depending on the exact location in the gene (and protein), the mutation may be putative of some key functional aberrancies that influence the IL-33 global functional properties (Figure 3). In general, IL-33 mutations occurred with very low frequencies in all tumors examined (0.072–1.391%). However, some mutations may putatively have a key impact on the functionality of IL-33. IL-33 protein is equipped with three main regions (Nuclear, Central, IL-1-like) with three specific binding and cleavage sequences in each of these domains (Figure 3). For example, there are 15 unique mutations in skin cancer patients, with a missense mutation (M52I at nucleotide 6250538 of the chromosome 9) targeting the chromatin binding site (R1). We hypothesize that this amino acid change potentially breaks the ability of IL-33 to bind DNA and thus to exert its regulatory functions. There are additionally 11 unique somatic missense mutations all affecting the IL-1-like domain of the protein (but outside the R3 sequence), that can putatively compromise the IL-33 binding ability to its specific receptor ST2 (3, 157). Another tumor that displays a mutation affecting the ability of IL-33 to bind the chromatin is the uterine cancer with the R48H amino acid change (Figure 3). Interestingly, the mutations E121K, H221Y, S225F, and H246Y, all occurring in the IL-1-like domain, are classified as deleterious in terms of IL-33 functional changes for skin cancers as evidenced by the cBioPortal database. Some of these mutations, like the amino acid change H221Y, are also shared with other tumors such as bladder cancer, thus denoting their significance. There is only one somatic missense alteration (G176S) occurring inside the R3 region, shared by colon and blood cancer patients (Figure 3). These mutations can putatively disrupt the cleavage site for caspase 3 and 7, thus increasing the IL-33 resistance to these proteases with key consequences in skin tumor survival and progression. Mutations in the cleavage site for inflammatory proteases (R2) occur only in colon and uterine tumors, with the A95V amino acid change (colon) and two mutations in uterine tumor (missense V101L and truncation S111).

**Figure 4:**
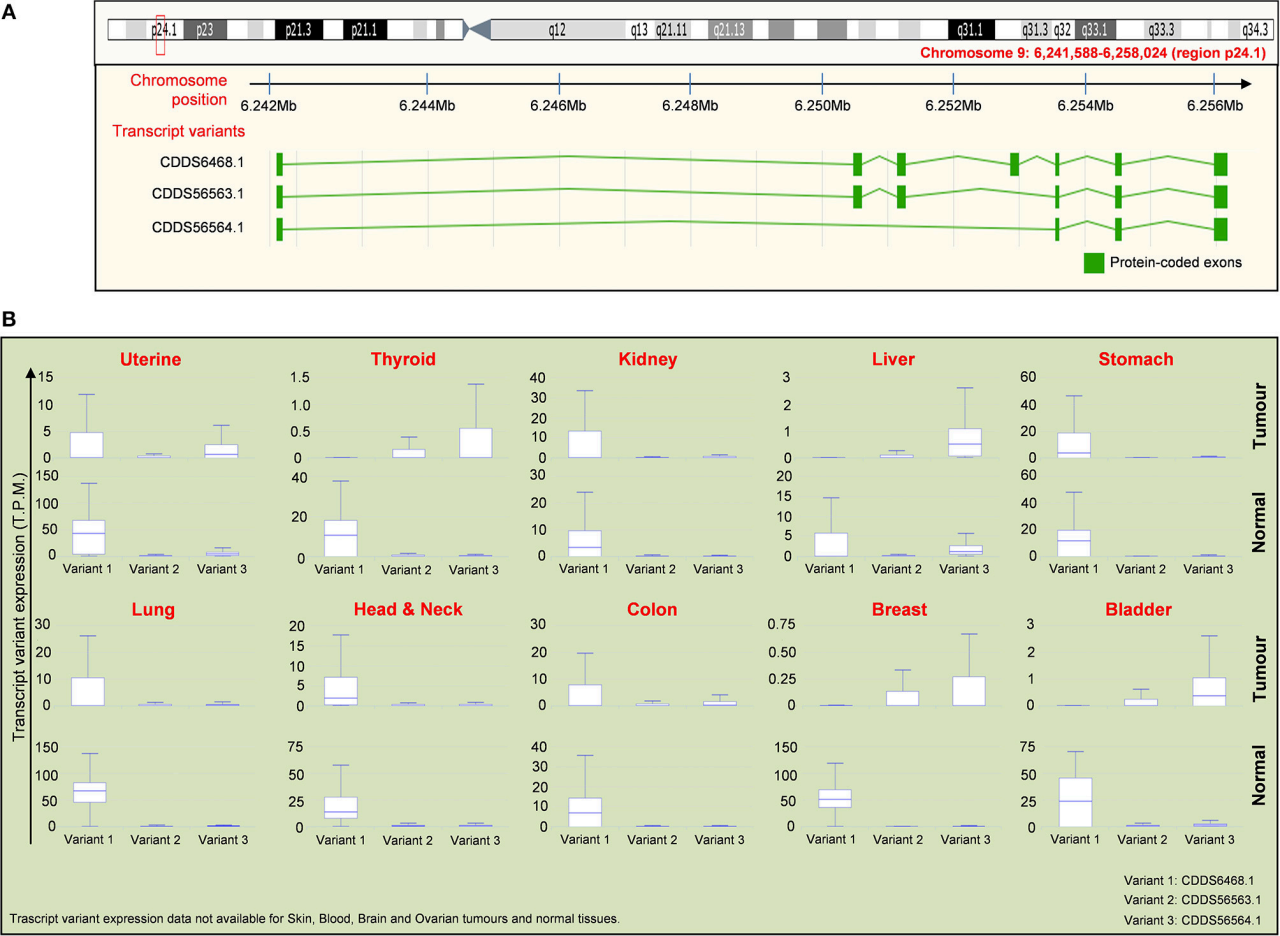
Distribution of IL-33 RNA transcript variants in tumor samples compared to normal tissues. **(A)** Schematic representation of the chromosome location (red box and text) of the IL-33 gene (upper panel) with the location of exons present in each indicated transcript variant (lower panel). Data are obtained by ENSEMBL genome browser (https://www.ensembl.org/). There are 3 isoforms of IL-33 gene, which are: NM_001199640 (CDDS56563.1), NM_001199641 (CDDS56564.1), NM_033439 (CDDS6468.1). **(B)** Expression of the IL-33 transcript variants indicated in **(A)** for each tumor compared to the normal tissues. Expression data (T.P.M, transcript per million) are obtained by interrogating the public database for Isoform expression resource analysis (Isoexpresso, http://wiki.tgilab.org/ISOexpresso/main.php). IL-33 transcript variant expression data are not available for skin, blood, brain, and ovarian cancers/normal tissues within Isoexpresso database. The predominant forms for brain and ovarian tumors are variants 1 whereas the predominant form expressed in skin tumors is the transcript variant 3 (resource: Mammalian Transcriptomic Database, http://mtd.cbi.ac.cn/).

As mentioned above, the IL-33 gene is equipped with a splicing system generating three different variants of the IL-33 protein (Figure 4A). The first isoform variant is composed by 7 exons, whereas the variants 2 and 3 contain 6 and 4 exons, respectively. The role and the extent of expression of each isoform in cancer is still a field of debate. Nevertheless, public databases such as IsoExpresso are now available, containing expression data of isoforms associated to thousands of human genes involved in cancer progression (158). We then extracted data about the expression of IL-33 isoform variants in different types of tumors and the normal counterparts (Figure 4B). The IsoExpresso database revealed a differential expression across the lesions and normal tissues or across the isoform variants of IL-33. Thyroid, liver, breast, and bladder cancers display a change in isoform expression when compared to normal counterparts. Indeed, variants 2 and 3 are highly expressed in these tumors compared to expression levels observed in normal tissues (Figure 4B). Of note, bladder cancer presents a splice site mutation (X157, Figure 3), and the presence of this mutation may probably be associated to the observed expression shift of the IL-33 isoform variants in this type of cancer (Figure 4). Indeed, in the tumor lesions the mainly expressed isoforms are variant 2 and 3, whereas the normal tissues express the variant 1 only (Figure 4B). This strongly suggests that this mutation affects the disappearing of IL-33 exon 4 (Figure 4A). In contrast, uterine tumor has been associated with a splice mutation (X115, close to the S111 truncation, Figure 3) not affecting the expression of IL-33 splice variants (Figure 4B).

## Concluding remarks

The role of IL-33 in tumorigenesis and cancer immunity remains controversial. The current literature indicate that IL-33 expression can be regulated during the progression of distinct types of cancers and that IL-33/ST2 signaling within the tumor microenvironment may differently contribute to tumorigenesis, promoting antitumor responses or mediating tumor growth or metastasis, depending on the nature of the malignant tissue. Further studies are needed to elucidate the role of this pathway at specific time points during cancer development as a possible diagnostic/prognostic marker for patients and to clarify whether IL-33/ST2 blockade may represent a valid approach for adjuvant therapies of established IL-33-dependent tumors.

It has been suggested that IL-33 has opposing effects in tumor immunity depending on its local concentration (121). In this respect, the tumor histotype may crucially determine the amount of IL-33 expressed. In addition, the presence of different IL-33 isoforms may play a crucial role. Thus, it may be possible that under steady-state conditions the homeostatic release of IL-33 by apoptotic epithelial cells may be regulated by caspase cleavage, thus limiting excessive inflammation. We envisage that, as in several chronic inflammatory diseases, also in tumors IL-33 could exist in several different forms as a result of post-translational processes, including intracellular and extracellular modification, or as a result of mRNA alternative splicing. There is compelling evidence that different forms and cleavage products of IL-33 can exert dissimilar biological activities. Unfortunately, the identification and the concentrations of the different forms of IL-33 in different tumors is largely unknown. Future studies should characterize the different forms of IL-33 in human and experimental tumors. Moreover, the mutation level of IL-33 gene observed in human solid tumors (Figure 3) may be associated with different isoforms and distinct stimulated antitumor immune responses. Lastly, since the new frontier of cancer immunotherapy is the employment of immune checkpoint inhibitors (i.e., against PD-1, PD-L1, CTLA-4) further studies are needed to clarify whether IL-33 conditioning may increase the therapeutic response to checkpoint blockade in cancer patients.

## Author contributions

GS, CA, and FM have conceived and designed the review. CA, CB, SA, MG, GV, GM, FM, and GS contributed intellectually and to the writing of the submitted version of the manuscript.

### Conflict of interest statement

The authors declare that the research was conducted in the absence of any commercial or financial relationships that could be construed as a potential conflict of interest.
